# Recent Advances in the Management of Chronic Hepatitis B

**DOI:** 10.5812/kowsar.1735143X.657

**Published:** 2011-08-01

**Authors:** Soo Ryang Kim, Jisin Yang, Masatoshi Kudo, Okio Hino

**Affiliations:** 1 Department of Gastroenterology, Kobe Asahi Hospital, Kobe, Japan; 2 Department of Gastroenterology and Hepatology, Kinki University School of Medicine, Osakasayama, Japan; 3 Department of Pathology and Oncology, Juntendo University, Tokyo, Japan

**Keywords:** Hepatitis B virus, Interferon alpha, Nucleotide analogues, Entecavir, Tenofovir

## Abstract

There are seven approved treatments for adults with chronic hepatitis B virus infection in the United States and European countries: interferon-α, pegylated interferon-α, lamivudine, adefovir dipivoxil, entecavir, telbivudine, and tenofovir disoproxil fumarate. At present, two new analogues, entecavir and tenofovir are recommended as the first line therapy by the guidelines of European Association for the Study of the Liver and American Association Study for the Liver Diseases. On the other hand, regarding interferon therapy, use of pegylated interferon-α is recommended as the first line therapy instead of standard interferon-α by both guidelines. Therefore, the main scientific interests and unmet medical needs for treatment of chronic hepatitis B have been narrowed down to long-term efficacy and safety of the two said analogues-entecavir and tenofovir-and combination therapy of pegylated interferon-α with the two analogues. To put it concretely, further studies are needed to assess (1) the long-term efficacy and safety and resistance to entecavir and tenofovir; (2) the efficacy of different durations (24 weeks to 2 years) and lower doses of pegylated interferon-α; (3) the role of combination therapy with two analogues to reduce resistance; and (4) the efficacy and safety of the two analogues with decompensated cirrhosis. Herein, we review the recent available data and results.

## 1. Introduction

There are seven approved treatments for adults with chronic hepatitis B (CHB) in the United States and European countries: interferon (IFN) α, pegylated (PEG) IFN-α, lamivudine (LAM), adefovir dipivoxil (ADV), entecavir (ETV), telbivudine (TBV), and tenofovir disoproxil fumarate (TDF). IFN-α and LAM have been approved for children with hepatitis B virus (HBV) infection. Two different treatment strategies are applicable in both HBeAg-positive and negative CHB patients: treatment with PEG IFN-α and long-term treatment with NUCs. There are several treatment options for patients, making rational choices for the first and second line treatment sometimes difficult. Although available randomized controlled trials show encouraging short-term results demonstrating a favorable effect of these agents on intermediate markers of the disease such as HBV DNA level, liver enzyme tests, and liver histology, limited rigorous evidence exists demonstrating the effect of these therapies on important long-term clinical outcomes, such as the development of hepatocellular carcinoma or a reduction in mortality rate. Questions therefore remain about which groups of patients benefit from therapy and at which point in the course of disease this therapy should be initiated.

Herein, we pooled the available data focusing on long-term efficacy and safety of two new analogues-entecavir and tenofovir-and combination therapy of PEG INF-α and the said two analogues. In the first section, we summarize recent findings based on the consensus of the guidelines of the European Association for the Study of the Liver (EASL) and the American Association Study for the Liver Diseases (AASLD) [1][2]. In section two, presentations at EASL and AASLD annual meetings in 2010 are reported.

## 2. Section I: Published Results Based on the Consensus of EASL and AASLD Guidelines

### 2.1. Assessment of Outcomes

Although various monitoring practices have been recommended, no clear evidence exists for an optimal approach. One proposed that the management algorithm used during therapy involves measuring HBV DNA and ALT levels every 12 weeks and HBeAg or anti-HBe levels every 24 weeks in patients who are HBeAg-positive. For patients who are HBeAg-positive and achieve a complete response (undetectable HBV DNA), seroconversion to anti-HBe may offer the opportunity to discontinue therapy after 6-12 months of "consolidation." During this period, regular monitoring of HBV DNA and HBeAg status should be done because relapse remains a possibility. HBsAg should be checked at 6-month intervals after HBe seroconversion if HBV DNA is undetectable. Quantitative HBsAg assay is still a research tool. HBeAg-negative patients should be similarly monitored for efficacy and safety through 48 weeks of treatment. A virological response with HBV DNA < 2000 IU/mL (approximately 10,000 copies/mL), i.e. 3.3 log10 IU/mL, is generally associated with remission of the liver disease. Undetectable HBV DNA in real-time PCR is the ideal desired of treatment sustained response with a high probability of HBsAg loss in the longer term. HBsAg should be checked at 6-month intervals if HBV DNA is undetectable. All patients treated with PEG IFN-α should be monitored for the known adverse effects of IFN. The balance of benefits and harms associated with screening for hepatocellular carcinoma is unknown and is an area for future research.

### 2.2. Antiviral Effect of NUCs

Table 1 sumarizes the efficacy of NUCs treatment in a 48-week large randomized controlled trial with HBeAg-positive and -negative patients.

**Table 1:** Summary of NUCs Treatment in Patients with HBeAg-Positive and -Negative Chronic Hepatitis B: 48 Weeks Post-Treatment Results

**Treatment Group**	**HBeAg Status**	**HBV DNA Suppression (log10 copies/mL)**	**HBV DNA Undetectable, %**	**ALT Normalization %**	**HBeAg Serocon- Version %**
LAM a [3][4]	Positive	- 5.4	36	60	18
	Negative	- 4.5	72	71	―
ADV a [5][6]	Positive	- 3.5	21	48	12
	Negative	- 3.9	51	72	―
ETV a [3][4]	Positive	-6.9	67	68	21
	Negative	- 5.0	90	78	―
TBV a [7]	Positive	- 6.4	60	77	23
	Negative	- 5.2	88	74	―
TDF a [8]	Positive	- 4.5 (12w)	76	68	21
	Negative	- 3.0 (12w)	93	76	―

^a^ Abbreviations: ADV, Adefovir; ETV, Entecavir; LAM, Lamivudine; TDF, Tenofovir; TVB, Telbivudine

2.2.1. Lamivudine (LAM)

In large registration trials, both on HBeAg-positive and -negative patients with CHB and those with previous IFN failure, a daily dose of 100 mg of LAM was compared to 0.5 mg of ETV. LAM treatment for 48 weeks resulted in suppression of HBV DNA by an average of 5.4 log10 copies/mL in HBeAg-positive patients and 4.5 log10 copies/mL in HBeAg-negative patients. HBeAg seroconversion occurred in 18% of patients, rendered HBV DNA undetectable (<102 copies/mL) in 36% (HBeAg-positive) to 72% (HBeAg-negative) of patients [3][4].

2.2.2. Adefovir (ADV)

In 48-week registration trials, CHB patients who were HBeAg-positive and -negative received 10 mg/day of ADV. ADV suppressed HBV DNA by 3.5 log10 copies/mL in HBeAg-positive patients and 3.9 log10 copies/mL in HBeAg-negative; HBV DNA decreased to an undetectable level (< 102 copies/mL) in only 21% of HBeAg-positive patients and to 51% of HBeAg-negative patients; suppression of HBV DNA was relatively slow; it was also less likely to induce HBeAg seroconversion (12%) [5][6].

2.2.3. Entecavir (ETV)

A daily dose of 0.5 mg of ETV was found to be superior to 100 mg of LAM in terms of suppression of HBV DNA by 6.9 log10 copies/mL in HBeAg-positive patients and by 5.0 log10 copies/mL in HBeAg-negative patients. Therapy with ETV was more likely to decrease HBV DNA to undetectable levels (< 102 log10 copies/mL) than LAM in 67% of HBeAg-positive patients and in 90% of HBeAg-negative of patients. Histological improvement was achieved in 72% of ETV-treated patients compared to 62% in LAM-treated patients (HBeAg positive); and in 70% of ETV-treated vs. 61% of LAM-treated patients (HBeAg-negative). The two drugs, however, did not differ in rates of HBeAg seroconversion-21% vs. 18%. Treatment effects were maintained with long-term ETV therapy, with HBeAg seroconversion rates increasing progressively to 31% at year two and 39% at year three. In addition, at the end of year two, HBsAg loss was recorded in 5% of ETV-treated and 2% of LAM-treated patients [9][10]. In the study of 96 weeks of treatment with o.5 mg of ETV in naïve Japanese patients, resistance was reported in only 1.7% [11].

2.2.4. Telbivudine (TBV)

TBV is a potent L-nucleoside that is believed to cause chain termination and is highly potent against HBV in cell culture. TBV (600 mg/day) was superior to LAM (100 mg/day) in suppressing HBV DNA to undetectable levels of < 102 copies/mL (60% vs. 40%; a reduction from 6.4 log10 to 5.5 log10 copies/mL), and in achieving histological improvement (65% vs. 56%) but not in normalization of ALT (77% vs. 75%) or serological responses (HBeAg seroconversion in 23% vs. 22%). In HBeAg-negative patients, TBV (600 mg/day) was superior to LAM (100 mg/day) in suppressing HBV DNA to undetectable levels (88% vs. 71%; reduction from 5.2 log10 to 4.4 log10 copies/mL) but not in achieving histological (67% vs. 66%) or normalization of ALT (74% vs. 79%) [7]. These responses were well maintained during the second year of therapy, and HBeAg seroconversion increased to 30% by the end of year two [12].

2.2.5. Tenofovir (TDF)

In two 48-week randomized controlled trials, oral TDF (300 mg/day) was compared to ADV (10 mg/day) in treatment-naive patients with HBeAg-positive and -negative CHB. In HBeAg-positive patients, TDF reduced HBV DNA levels by 4.5 log10 IU/mL (12 weeks results) and suppressed HBV DNA to undetectable levels (< 102 IU/mL) in 76% of patients vs. in only 13% in the ADV group. TDF and ADV treatments resulted in similar rates of histological benefit (74% vs. 68%) and HBeAg seroconversion (21% vs. 18%). An important finding was HBsAg loss in 3% of patients during the first 48 weeks of therapy in the TDF group [8]. In the HBeAg-positive group, at the end of year two of continuous TDF treatment, HBeAg seroconversion increased to 27% and HBsAg loss increased to 6% [13]. In HBeAg-negative patients, TDF reduced HBV DNA levels by 3.0 log10 IU/mL (12 weeks results) and suppressed HBV DNA to undetectable levels (< 102 IU/mL) in 93% of patients vs. in only 63% in the ADV group.

### 2.3. Antiviral Resistance to NUCs

Frequencies of antiviral resistance within five years of administration for the five NUCs are shown in Table 2. Although LAM has the most extensive safety record, its current use is limited by the high frequency of LAM resistance (24% in year one, and 70% in year four) [14], and the availability of more potent agents with superior efficacy and markedly improved resistance profiles.

**Table 2:** Frequency of Antiviral Resistance to Nucleoside Analogs Treatment

**Treatment Group **	**Treatment Duration, y**	**Antiviral-resistance, %**
LAM a [14]	1	24
	2	42
	3	53
	4	70
ADV a (naive patients) [6]	1	0
	2	3
	3	11
	4	18
	5	29
ETV a (naive patients) [10]	4	< 1
TBV a (naive patients) [7][12]	1	2-5
	2	11-25
TDF a [15]	2	0

^a^ Abbreviations: LAM, Lamivudine; ADV, Adefovir; ETV, Entecavir; TVB, Telbivudine; TDF, Tenofovir

ADV is more expensive than TDF, is less effective, and produces higher rates of resistance. Although resistance to ADV is slow to emerge, resistant variants increase progressively after the first year, reaching 29% in year five [16]. The advantages of ADV are its limited resistance during first two years, the absence of cross-resistance with LAM and other L-nucleosides and, therefore, its value as treatment for LAM-resistant CHB [17][18] and for hepatic decompensation associated with LAM resistance prior to and after liver transplantation [19]. The high potency and excellent safety profile of ETV are complemented by its very high barrier to resistance in treatment-naive patients (< 1%) in year four. ETV and TDF are potent HBV inhibitors and they have a high barrier to resistance [3][20][21]. Therefore, they can be confidently used as the first-line monotherapies. The role of monotherapy with ETV or TDF could be modified if higher rates of resistance become apparent with longer treatment duration. In a study of 96 weeks of treatment with TDF, no evidence of TDF resistance was found [15].

TBV is a potent inhibitor of HBV but, due to a low genetic barrier to resistance, a high incidence of resistance has been observed in patients with high baseline levels of replication and in those with detectable HBV DNA after 24 weeks of therapy. In a large registration trial, TBV was compared to LAM in HBeAg-positive and -negative patients. The frequency of antiviral resistance to TBV at one year was 5% of HBeAg-positive and in only 2% of HBeAg-negative patients [7].

Virological breakthrough in compliant patients is related to viral resistance. Resistance is associated with prior treatment with NUCs (i.e., LAM, ADV, TBV, emtricitabine) or, in treatment-naive patients, with high baseline HBV DNA levels, a slow decline in HBV DNA and partial virological response during treatment. Resistance should be identified as early as possible before clinical breakthrough (i.e. increased ALT) by means of HBV DNA monitoring; if possible, the pattern of resistance mutations should be identified to adapt therapeutic strategies. Indeed, clinical and virological studies have demonstrated the benefit of an early treatment adaptation-as soon as viral load increases [22][23]. In case of resistance, an appropriate rescue therapy should be initiated with the most effective antiviral effect and the minimal risk to induce multiple drug-resistant strains. Therefore, adding-on a second drug without cross-resistance is the only efficient strategy.

-LAM resistance: Add TDF (add ADV if TDF is not yet available).

-ADV resistance: It is recommended to switch to TDF if available and add a second drug without cross-resistance. If an N236T substitution is present, add LAM, ETV or TBV or switch to TDF plus emtricitabine. If an A181T/V substitution is present, add ETV (the safety of the TDF-ETV combination is unknown) or switch to TDF plus emtricitabine.

-TBV resistance: Add TDF (add ADV if TDF is not yet available). The long-term safety of these combinations is unknown.

-ETV resistance: Add TDF (the safety of this combination is unknown).

-TDF resistance: Resistance to TDF has not been described so far. It is recommended that genotyping and phenotyping be done by an expert laboratory to determine the cross-resistance profile. ETV, TBV, LAM or emtricitabine could be added (the safety of these combinations is unknown).

### 2.4. Long-term Therapy with NUCs

HBV DNA levels should be monitored at week 12 to ascertain virological response and then every 12 to 24 weeks. HBV DNA reduction to undetectable levels by real-time PCR (i.e. < 10-15 IU/mL) should ideally be achieved to avoid resistance. HBV DNA monitoring is thus crucial to detect treatment failure. In HBeAg-positive patients, HBeAg and subsequently anti-HBe antibodies once HBeAg is negative should be measured at intervals of 6 to 12 months. NUCs are cleared by the kidneys, and appropriate dose adjustments are recommended for patients with reduced creatinine clearance. Drug concentrations are comparable in patients with varying degrees of hepatic impairment but this has not been fully studied. Exacerbations of hepatitis B may occur and require more intensive monitoring (monthly in the first three months) in patients with cirrhosis. The onset of complications in these patients requires urgent management. Renal impairment has rarely been reported in patients with HIV infection receiving anti-HBV drugs, or in patients receiving nephrotoxic drugs and treated with TDF or ADV, thus, appropriate monitoring for nephrotoxicity and dose adjustments are necessary.

Long-term monitoring for carcinogenesis with ETV is ongoing. Myopathy has rarely been reported in CHB patients treated with TBV. Peripheral neuropathy has been observed in patients treated with PEG IFN and TBV and thus this combination should be avoided.

### 2.5. Treatment with PEG IFN-α

The main theoretical advantages of IFN-α (conventional or PEG) are the absence of resistance and the potential for immune-mediated containment of HBV infection with an opportunity to obtain a sustained virological response off-treatment and a chance of HBsAg loss in patients who achieve and maintain undetectable HBV DNA. Frequent side effects and subcutaneous injection are the main disadvantages of IFN-α treatment. IFN-α is contraindicated in patients with decompensated HBV-related cirrhosis or autoimmune disease and in those with uncontrolled severe depression or psychosis. Full information about the advantages, adverse events and inconveniences of PEG IFN-α vs. NUCs should be provided so the patient can participate in the decision. There has been a resurgence of interest in IFN therapy the past five years, largely based on results of large clinical trials demonstrating that PEG IFN has more potent antiviral activity than standard IFN-α and that in contrast to NUCs, it does not result in any antiviral resistance and can be given for a finite period rather than indefinitely. Therefore, when compared to the standard IFN α-2a in a dose of 4.5 million units three times weekly, PEG IFN in a dose of 180 μg once weekly for 12 months resulted in a greater decline in HBV DNA levels and a higher rate of HBeAg seroconversion (33% vs. 25%) [24]. Three large multicenter trials of PEG IFN therapy have been published-two in HBeAg-positive [25][26] and one in HBeAg-negative CHB patients [27]. Each study included treatment arms in which PEG IFN was used alone or in combination with LAM. Two studies used PEG IFN α-2a and one used PEG IFN α-2b. The results 24 weeks after treatment are shown in Table 3.

**Table 3:** Summary of Combination Therapy of PEG INF and LAM in CHB Patients 24 Weeks Post-treatment

**HBeAg Status**	**Treatment Arms**	**No.**	**HBV DNA Suppression, %**	**HBV DNA Undetectable, %**	**ALT Normalization, %**	**HBeAg Seroconversion, %**
Positive [25]	PEG IFN 100 μg/wk × 32 wk→ 50 μg × 20 wk	136	27	7	32	29
	PEG IFN 100 μg/wk × 32 wk→ 50 μg × 20 wk + LAM	130	32	9	35	29
Positive [26]	PEG IFN 180 μg/wk × 48 wk	271	32	14	41	32
	PEG IFN 180 μg/wk + LAM 48 wk	271	34	14	39	27
	LAM for 48 wk	272	22	5	28	19
Negative [27]	PEG IFN 180 μg/wk × 48 wk	177	43	19	59	―
	PEG IFN 180 μg/wk + LAM 48 wk	179	44	20	60	―
	LAM for 48 wk	181	29	7	44	―

In a multinational European study, PEG IFN α-2b was given in a dose of 100 μg weekly for 32 weeks followed by 50 μg weekly until completion of 52 weeks of treatment with or without LAM (100 mg daily) in 266 patients who were HBeAg-positive [25]. Seroconversion of HBeAg by six months after treatment occurred in similar proportions of patients receiving monotherapy as combination therapy (29% vs. 29%) as did loss of HBsAg (7% vs. 7%). Suppression of HBV DNA levels and loss of HBeAg were greater on combination therapy than monotherapy, but relapse rates were higher in the group that received LAM so that sustained responses six months after stopping treatment were equivalent. A comparison group receiving LAM alone was not included. In a second larger multicenter trial, a total of 814 patients with HBeAg-positive CHB were given either PEG IFN α-2a alone (180 μg once weekly), LAM alone (100 mg daily), or the combination for 48 weeks [26]. Again, HBV DNA suppression was greater in patients receiving combination therapy than in those receiving either PEG IFN or LAM monotherapy. However, rates of HBeAg seroconversion six months after discontinuation of therapy was greater with PEG IFN than LAM monotherapy (32% vs. 19%) and was no higher with combination therapy (27%). Loss of HBsAg occurred in 16 of 542 (3%) patients who received PEG IFN (alone or with LAM) but in none of 272 patients receiving LAM alone (P = 0.004).

Finally, in another large multicenter trial, patients with HBeAg-negative hepatitis B were treated with PEG IFN α-2a alone (180 μg once weekly), LAM alone (100 mg daily), or the combination for 48 weeks [27]. Six months after stopping therapy, the percentage of patients with normal ALT values or HBV DNA levels below 20,000 copies/mL was significantly higher with PEG IFN monotherapy (59% and 43%, respectively) than with 48 weeks of LAM monotherapy (44% and 29%, respectively). Again, the addition of LAM to PEG IFN did not appear to increase the response rates even though there was greater HBV DNA suppression in combination therapy. Furthermore, 3% (12/356) of patients who received PEG IFN but none of 181 patients who received LAM alone became HBsAg-negative.

These three studies showed that a one-year course of PEG IFN induced HBeAg seroconversion in about one-third of HBeAg-positive patients and induced a lasting biochemical and virological response in almost 40% of HBeAg-negative patients. Furthermore, therapy with PEG IFN led to loss of HBsAg in a small proportion of patients, an outcome not seen with a one-year course of LAM therapy. Adding LAM to PEG IFN did not increase the rate of sustained responses. These results suggested that a trial of one-year course of PEG IFN might be appropriate in selected patients with CHB, before embarking on long-term suppressive therapy with NUCs.

## 3. Section II. Topics of EASL and AASLD Presentations at Annual Meetings in 2010

### 3.1. Efficacy of NUCs

3.1.1. EASL Abstract 1009

Effectiveness of ETV for NUC-naive HBeAg-negative CHB patients in clinical practice: A two-year multicenter cohort study on 311 patients. Lampertico P.

3.1.1.1. Key Results

311 consecutive NUC-naive HBeAg-negative CHB patients, recruited in 17 Italian liver units, were treated with ETV 0.5 mg for 23 months [10][28].

-294 (94%) patients achieved a virological response (97% at week 48).

-Two patients had primary non-response at week 12, and three (1%) patients had a virological breakthrough. No ETV resistance in two patients and suboptimal compliance in one patient.

-Two (0.6%) patients cleared HBsAg, seroconverted to anti-HBs and stopped ETV.

-No ETV-related serious adverse events were reported.

-19 (6%) patients had a partial virological response at week 48; 50% with HBV DNA > 1000 IU/mL. TDF + ETV inhibited HBV replication in the high viral load partial responders.

3.1.1.2. Comments

This cohort supports ongoing evidence that ETV was effective in this real life population of primarily HBeAg-negative patients, of whom almost 50% were with LC.

3.1.2. AASLD PO 391

Maintained long-term suppression of HBV replication in NUC-naive patients with CHB treated with ETV monotherapy in field practice: The Italian multicenter experience. Lampertico P, et al.

-Virological responses increased over time in both HBeAg-positive and -negative patients, with more than 90% achieving undetectable HBV DNA.

-HBV remained suppressed over time in the vast majority of patients; only 4% of patients showing a short lasting virological blip.

-Serological responses, i.e. HBeAg seroconversion and HBsAg loss, increased over time. Five patients stopped ETV successfully. And most patients developed a normal ALT level.

-Renal safety: Serum creatinine increased in few patients (< 1%)-an event not considered drug-related.

-Patient retention rates were 84%.

3.1.2.1. Comments

This reconfirms ETV monotherapy suppresses HBV replication in most NUC-naive patients in real practice up to 30 months, independently of serology and safety profile was consistent with registration studies.

3.1.3. AASLD PO 369

Effectiveness and safety of TDF in field practice: A multicenter European cohort study of 737 patients with CHB. Lampertico P, et al.

-Most NUC-naive patients achieved undetectable HBV DNA by PCR assay and developed a normal ALT level. Viral suppression was however significantly faster in patients with lower baseline viremia.

-Primary non-response at week 12 and partial virological response at week 48 occurred in 3% and 18% of the patients, respectively.

-Eight patients seroconverted to anti-HBe with an 18-month cumulative probability of 32% and two cleared HBsAg.

-In NUC-experienced patients, HBV DNA became undetectable in almost 74% of the patients, independently of treatment regimen (TDF vs. TDF + LAM).

-No major changes of renal function (glomerular and tubular) were observed over 18 months of treatment. Dose adjustments, hypophosphatemia and increased phosphate wasting occurred more frequently in NUC-experienced patients.

3.1.3.1. Conclusions

TDF suppressed HBV replication in most NUC-naive and NUC-experienced patients in field practice up to 18 months. The safety profile was favorable with few patients, mainly NUC-experienced, showing some degrees of renal dysfunction.

3.1.3.2. Comments

This adds another clinical data of TDF. Data on renal toxicity seem not to be consistent. Therefore, we will wait and see the accumulation of more data.

### 3.2. Resistance Data

3.2.1. AASLD PO 1365

No resistance to TDF was detected in following up to 192 weeks of treatment in patients mono-infected with CHB virus. Snow-Lampart A, et al. This evaluated NUC-naive, 176 HBeAg+ and 250 HBeAg- patients on TDF treatment up to four years. No resistance was detected in 348 patients at 192 weeks. Some doctors chose to add emtricitabine (FTC) 200 mg to TDF for seven patients from the 3rd year and five from the 4th year on, if patients were confirmed to be viremic at week 72 or beyond. They reported good tolerability of TDF.

3.2.1.1. Comments

TDF is shown to be free from resistance in this clinical trial setting. It needs to be examined in real practice usage. (cf. ETV has been presented to have 1.2% resistance rate in five years as shown in 2009 EASL guidelines).

### 3.3. Safety Evaluation of NUCs

3.3.1. Long-term Data

3.3.1.1. EASL Poster 1016

Low rates of nucleos(t)ide-associated adverse events in the long-term experience with ETV. Manns M, et al.

3.3.1.1.1. Key Results

Long-term safety data from the roll-over study ETV-901 are reviewed, focusing on adverse events (AEs) with a potential nucleos(t)ide association. Median exposure to ETV in ETV-901 was 184 weeks (almost 3.5 years) (Table 4). Of the 1,051 patients in this analysis, 448 (46%) had prior ETV exposure in previous studies. Overall, the most common AEs (related and unrelated) were upper respiratory tract infection (27%), headache (20%) and nasopharyngitis (16%). Lactate increase or bicarbonate decrease occurred in six (< 1%) patients and no cases of lactic acidosis syndrome were reported. Rates of serious AEs, discontinuations due to AEs, liver disease progression and ALT flares were consistent with previous Phase III observations. AEs typically associated with NUCs use were reported infrequently by investigators. Study ETV-901 demonstrates that ETV is generally a well-tolerated treatment at a dose of 1.0 mg/day when used to treat a diverse population of CHB patients.

**Table 4:** Adverse Event (AE) Results from 901 Studies (Mean of 184 Weeks of Treatment) (n=1051)

**Adeverse Events (AEs)**	**Total, No.(%)**
Any AEs	900 (86)
Serious AEs	169 (16)
Discontinuations due to AEs	14 (1)
Grade 3-4 AEs	203 (19)
Grade 3-4 AEs considered related to ETV	45 (4)
All deaths	27 (3)
Liver-related deaths	12 (1)
Non-liver-related deaths	15 (1)

3.3.1.1.2. Comments

This roll-over study ETV-901 provides an opportunity to assess safety events in a large cohort of diverse CHB patients, 1051 patients who received long-term ETV (1.0 mg/day) therapy in the study over a median of 184 weeks. Study ETV-901 demonstrates that ETV is a generally well-tolerated long-term treatment.

3.3.2. Renal Data

3.3.2.1. EASL Abstract 1007

Risk of renal toxicity with TDF for CHB. Gish R, et al.

3.3.2.1.1. Key Results

84 patients on TDF (either monotherapy or in combination with another antiviral drug) were matched by age (± 5 years) and gender to 84 ETV monotherapy patients.

-TDF was shown to be well tolerated: Serum creatinine increases of 0.2 mg/dL were found to be common, whereas such an increase was rare for the TDF arm and less than ETV group (2%, P = 0.029) probably due to a significantly higher rate of dose adjustments.

-History of diabetes, and transplant, significantly increased the risk of renal injury in all CHB patients (P = 0.004, and 0.002, respectively).

3.3.2.1.2. Comments

This is a presentation with the risk of TDF with nephrotoxicity. The study, however suffers from some limitations including, retrospective analysis of data that may cause a selection bias for patients with renal problems to be given ETV as TDF was given as monotherapy or in combination therapy. Patients on ETV had longer duration of disease and comorbidities were not equally matched between the study arms. Furthermore, dose of TDF were often adjusted.

3.3.2.2. EASL Abstract 1010

OPTIB study: A multicenter prospective open label study on TDF for CHB patients with suboptimal response to ADV or ADV+LAM treatment. Levrero M, et al.

3.3.2.2.1. Key Results

Adults with HBV monoinfection and HBV DNA > 103 copies/mL after 48 weeks of ADV with or without LAM were enrolled and switched to TDF 300 mg daily with or without LAM.

-91 patients were screened and 85 were enrolled. 13 (15%) patients were switched from ADV to TDF and 72 (85%) to TDF + LAM combination.

-The median duration of prior ADV therapy was 29.2 months.

-At 24 weeks of treatment, median HBV DNA fall from baseline was 2.02 log10 IU/mL and 62% had HBV DNA levels < 69 IU/mL and 49% HBV DNA levels < 12 IU/mL.

-At 48 weeks, 81% of patients had HBV DNA levels < 69 IU/mL and 65% had HBV DNA levels < 12 IU/mL.

-The proportion of patients reaching negativity through 48 weeks was not correlated with HBeAg status or the presence of ADV resistance mutations at the baseline.

-No clinically significant side effects related to TDF were reported.

3.3.2.2.2. Comments

Despite the concerned nucleotide cross-resistance profile, this study showed higher response rates than other presented data sets. This study implies that TDF can be used to salvage patients exposed to ADV and/or ADV + LAM.

3.3.2.3. EASL Abstract 1028

Renal safety and antiviral efficacy of TDF monotherapy in nucleos(t)ide analogue refractory patients with hepatitis B virus (HBV) mono-infection. vanBommel F, et al.

3.3.2.3.1. Key Results

Data from all HBV monoinfected patients treated with TDF monotherapy in 19 European centers were retrospectively analyzed. Of 343 patients screened, 195 were found eligible for retrospective data analyses; 137 were HBeAg-positive. The mean ± SD HBV DNA level was 6.9 ± 1.5 (range 4-10) log10 copies/mL.

-After 48 months of TDF therapy a mean decrease of estimated glomerular filtration rate (eGFR) of 9% was observed.

-During the total observation period, 10 patients had a moderate decrease (20%-30%) in eGFR; six patients had a severe decrease (> 30%), however eGFR remained within normal values in most patients and did not decrease to < 50 mL/min in patients with initially normal eGFR values.

-TDF dosage did not need to be adjusted due to changes in creatinine.

-A model assessing the influence of age on the eGFR rates as determined by the MDRD formula confirmed a mild decrease in eGFR driven by increase in serum creatinine during the 48 months.•

-A comparison of the mean eGFR rates in the TDF group and the control group showed no significant differences in eGFR decrease.

3.3.2.3.2. Comments

In this ongoing real world, independent cohort study evaluating TDF in refractory patients, it was shown that TDF is not associated with renal issues, though this study has excluded patients with higher risk of renal toxicity including, concomitant comorbidities-i.e. those with kidney disease, arterial hypertension, and/or diabetes.

3.3.2.4. AASLD PO 393

Prevalence of renal alterations indicative of proximal tubular damage (PTD) in patients with CHB virus infection during long-term therapy with TDF. vanBommel, et al.

3.3.2.4.1. Summary

In total, 24 of 61 (39%) patients showed at least one sign of PTD, which would be either renal phosphate loss (hypophosphatemia and/or TmPO4/GFR↓), glucosuria or increased α1-microglobulin/creatinine ratio.

3.3.2.4.2. Conclusion

This study confirms that long-term treatment with TDF does not lead to a significant decrease in eGFR in HBV-infected patients, regardless of age or risk factors for kidney dysfunction. However, signs of PTD were prevalent in 39% of patients after mean treatment duration of 29 months. As there were no samples from baseline available, there was no clear association between these alterations and the use of antiviral agents. Therefore, further follow-up data are needed to determine the role of TDF therapy in possible proximal tubular damage. More specific markers may help to further determine the drug's influence on renal function.

3.3.3. Bone Study

3.3.3.1. AASLD PO 414

High prevalence of reduced bone mineral density in patients with CHB under nucleos(t)ide analogues treatment. Vigano M, et al. Single center (Universita di Milano), cross-sectional study studied 319 patients with CHB receiving NUC over a one-year period. Dual X-ray absorptiometry (DEXA) of the lumbar spine (LS) and femoral neck (FN) revealed that two thirds of CHB patients undergoing NUC treatment had reduced bone mineral density (BMD), osteoporosis at either LS or FN was present in 19% and osteopenia in 49% of the patients. Multivariate analysis showed that female sex, older age and nucleotide (ADV and TDF) treatment were independently associated with a reduced BMD.

3.3.3.1.1. Comments

It is noteworthy that only nucleotides (ADV and TDF), not nucleosides (ETV and LAM), was associated with reduced BMD. Clinicians may need periodical screening of patients for osteoporosis.

3.3.4. PEG IFN for CHB

3.3.4.1. EASL Abstract 98

Extended (two years) treatment with PEG INF α-2a [40 kD] improves sustained response rates in genotype D patients with HBeAg-negative CHB. Lampertico P, et al.

3.3.4.1.1. Results

PEG IFN α-2a 180 µg/week was evaluated for HBeAg-negative patients with CHB (n = 128) for its duration (48 vs. 96 weeks) (Table 5). Virologic response was superior with 96 weeks and notably HBsAg loss retention was observed in 10% of patients one year after the therapy of 96 weeks. These edges NUCs in efficacy in this study population, but it should be noted that different genotypes would respond differently and we need further studies in patients with various backgrounds.

**Table 5:** Safety Profiles of Extended PEG IFN Therapy

**Safety Outcome**	**48-Week PEG IFN a (n = 51)**	**96-Week PEG IFN (n = 52)**
≥ 1 AE a, %	82	77
≥ 1 serious AE, %	14	6
Need for dose reduction, %	31	19
Study withdrawal, %		
Due to AEs	16	12
Reasons other than safety	8	12
Death b, No.	1	0

^a^ Abbreviations: AE, adverse event; PEG IFN, peginterferon

^b^ Patient died of hepatocellular carcinoma during follow-up

3.3.4.1.2. Comments

It is still in the experimental stage but this deserves to be examined further, although long-term treatment poses cost and safety concerns and may limit the number of eligible patients for this therapy.

### 3.4. Efficacy and Safety of NUCs in Decompensated Cirrhosis

Decompensated cirrhosis is a serious complication of CHB. The five-year survival of patients with decompensated cirrhosis (14%) has been reported and is lower than that for patients with compensated cirrhosis (84%) [2]. However, suppression of viral replication with antiviral therapy has been shown to result in clinical improvement and increased survival [2]. There are limited data on safety and efficacy of NUC therapy in patients with CHB and decompensated cirrhosis. Summarized here are recently presented data including two randomized clinical trials [28][29], and a cohort study on Korean patients pertaining to the use of ETV. in this patient population.

3.4.1. EASL Oral Abstract 7

Treatment of decompensated HBV-cirrhosis: results from a two-year randomized trial with telbivudine or lamivudine. Gane EJ, et al. (Table 6). This study was to evaluate clinical and virological outcomes of TBV vs. LAM in 232 patients (mean CTP and MELD score TBV 8.1 and 14.7; LAM 8.5 and 15.5). At baseline, the median age was almost 50 years-65% Asian, almost 73% males, and approximately 57% HBeAg-negative. This RCT showed that both therapies were safe but with high rates of rebounds/virological breakthroughs. There was only a limited improvement in MELD score of 0.2 with TBV, and 1 with LAM.

**Table 6:** Outcomes in telbivudine (TBV) and lamivudine (LAM) Groups

2-Year Outcome (ITT Population)	**TBV a (n = 114)**	**LAM a (n = 114)**	***P* value**
HBV DNA, % (< 300 copies/mL)	49	40	0.15
Viral breakthrough, % (HBV DNA > 1 log10 copies/mL above nadir)	28	37	0.16
Composite endpoint, %	34	24	0.004
CTP score improved or stabilized, %	75	74	NS a

^a^ Abbreviations: LAM, lamivudine; NS, not significant; TBV, telbivudin

3.4.1.1. Comments

In this large scale study with long-term follow-up, TBV was well tolerated, stabilized liver function and had comparable tolerability to LAM. Safety profiles were similar between treatment arms, however, both TBV and LAM showing almost 30% viral breakthrough. This result seems to reinforce the need to use potent antiviral treatment with low rates of resistance in this population with advanced disease, like ETV or TDF.

3.4.2. EASL Abstract 1011

Risk and predictors of mortality or hepatocellular carcinoma among ETV- or ADV-treated CHB patients with evidence of hepatic decompensation. Liaw Y, et al.

3.4.2.1. Key Results

This industry-sponsored study examined predictors of death and HCC in pooled data from ETV-treated and ADV-treated patients. The baseline predictors for death and HCC were examined in the 191 patients randomized to receiving either 1.0 mg/day ETV or 10 mg/day ADV for up to 96 weeks using univariate and multivariate Cox proportional hazard models with pooled data. Significant predictors of mortality in univariate analysis included serum creatinine level, MELD score, total bilirubin and albumin level. The multivariate analyses showed that a decreased hepatic function (increased bilirubin and decreased albumin level) is a significant predictor of mortality among CHB patients with decompensated liver disease treated with nucleos(t)ide analogues. Cumulative HCC rates were 12% and 20% among ETV-treated and ADV-treated patients, respectively. Cumulative death rates were 23% and 33% among ETV-treated and ADV-treated patients, respectively. HBV genotype B/C was the only predictor for development of HCC (Table 7).

**Table 7:** Cumulative Efficacy and Safety in Both entecavir (ETV) and adefovir dipivoxil (ADV) Groups

	**Week 48**	
	**ETV a**	**ADV a**
Cumulative efficacy of NUCs		
HBV DNA change from baseline, (log10 copies/mL)	4.66	3.90
HBV DNA, No. (%) (< 300 copies/mL)	57 (100)	20 (91)
HBeAg loss, No.%	11 (54)	18 (51)
HBeAg seroconversion, No.%	6 (54)	10 (51)
HBsAg loss, No.%	5 (100)	0 (91)
CTP score improvement or no worsening, No.%	61 (100)	67 (91)
CTP score, No.% ( ≥ 2-point reduction)	35 (100)	27 (91)
MELD score change from baseline, Mean (SE) a	2.6 (0.62)	1.7 (0.50)
Cumulative safety of NUCs		
Any AE, No.%	91 (89)	86 (97)
Grade 3-4 AEs, No.%	55 (54)	42 (47)
Deaths, No.%	23 (23)	29 (33)
Serum Cr, No.%, (≥ 0.5 mg/dL increase)	17 (17)	21 (24)
HCC, No.%	12 (12)	18 (20)
Discontinuation due to AEs, No.%	7 (7)	5 (6)

^a^ Abbreviations: ADV, adefovir dipivoxil; ETV, entecavir; SE, standard error

3.4.2.2 Comments

The ETV-048 subanalysis reinforces the importance of biologic risk factors (including baseline characteristics and inclusion criteria) as predictors associated with increased HCC and/or mortality in decompensated cirrhotic patients.

### 3.5. Cohort Study in Korean Patients

Shim JH et al. Efficacy of ETV in treatment-naive patients with HBV-related decompensated cirrhosis [30]. This cohort study evaluated the effect of ETV monotherapy (0.5 mg QD for ≥ 12 months) on viral suppression and hepatic function in 70 consecutive treatment-naive patients with HBV-associated decompensated cirrhosis (defined as CTP ≥ 7 [class B and C]), or the presence of portal hypertension complications). Comparator group consists of compensated LC patients with HBV (n = 144). Virologic response in this decompensated group (n = 55) was also compared to compensated cirrhosis. 15 patients in the decompensated group received ETV < 12 months and therefore were not included in the comparative analysis with the compensated group. The baseline characteristics for decompensated and compensated groups were similar for gender ratio, HBV DNA levels (mean ± SD for total patients was 7.34 ± 1.43 log10 copies/mL; n = 199), and proportion HBeAg-positive (mean for total patients: 58.8%; n = 199).

However, in comparison to the compensated group, those with hepatic decompensation had a greater mean age (52.6 vs. 46.8 year, P < 0.001), lower mean ± SD serum ALT (101.9 ± 110.7 vs. 156.5 ± 160.5 IU/L, P = 0.021); and higher mean ± SD CTP (8.1 ± 1.7 vs. 5.3 ± 0.05) and MELD (11.5 ± 3.9 vs. 7.0 ± 1.5) scores (P < 0.001 for both).

Virologic, serologic and biochemical responses after 12 months of ETV therapy in the decompensated and compensated groups are presented in Table 8. Overall, at 12 months the rates for undetectable HBV DNA, HBeAg loss/seroconversion, and ALT normalization were not significantly different between the compensated and decompensated groups. In an intention-to-treat analysis of efficacy of all 70 patients with decompensated cirrhosis, the cumulative rates of HBV DNA undetectability and HBeAg loss at 12 months were 92.3% and 54.0%, respectively. For those patients with decompensated cirrhosis (n = 70), the cumulative incidence of HCC was 6.9% at month 24; four patients developed HCC during the follow-up. The cumulative incidence of mortality or OLT was 12.9% at month 12 and 17.0% at month 24. For 55 patients with decompensated liver function treated with ETV for ≥ 12 months, improvements from baseline in CTP score and its components (albumin, total bilirubin, prothrombin time) and MELD score were observed (P < 0.05 for all). CTP class A (score 5 or 6) was achieved in 65.5% [30] of the patients, and improvement in CTP (≥ 2 points reduction) was observed in 49% [27] of the patients after 12 months of treatment.

**Table 8:** One-year Results of Virologic and Biochemical Indices

One-year Results	**Compensated (n = 144)**	**Decompensated (n = 55)**	***P***** value**
Change in HBV DNA, (log10 copies/mL)	6.74 ± 1.88	6.82 ± 1.32	0.793
HBV DNA undetectable, No. (%), (< 300 copies/mL by PCR)	113/144 (78.5)	49/55 (89.1)	0.104
HBeAg seroconversion, No. (%)	22/90 (24.4)	6/27 (22.2)	0.812
HBeAg loss, No. (%)	37/90 (41.1)	13/27 (48.1)	0.517
ALT normalization, No. (%)	108/144 (75.0)	42/55 (76.4)	0.535

3.5.1. Comments

This cohort study supports the use of ETV as a first-line treatment option for NUC-naive patients with decompensated HBV cirrhosis. Further follow-up of similar studies are needed to identify the optimal treatment for these patients and those with LAM-resistant HBV cirrhosis.
